# The Diabetic Nephropathy and the Development of Hypertension in Rats

**DOI:** 10.1155/EDR.2001.195

**Published:** 2001

**Authors:** Adriana Zuccollo, Monica Navarro, Orlando Catanzaro

**Affiliations:** 1 PROSIVAD-CONICET Catedra de Animales de Laboratorio Facultad de Farmacia y Bioquimica-UBA Argentina; 2 PROSIVAD-CONICET Instituto Universitario de Ciencias de la Salud Facultad de Medicina-Fundacion Barcelo Argentina; 3 Universidad Del Salvador Fac. de Medicina Cat. de Fisiologia Tucuman Buenos Aires 1845 CP 1050 Argentina

## Abstract

The present study was designed to examine the development of hypertension in diabetic rats treated with streptozotocin (STZ, 1mg/g bw). The rats were studied at 3, 6, 9, 12 and 15 weeks. From the third week the rats were divided in diabetic rats according their glycemias and controls, along 15 weeks. After the third week a group, of rats showed increased urinary protein excretion (93, 134, 155 and 191%) compared to controls. In this group of rats the urinary kallikrein excretion was lower than control and the systolic blood pressure became significantly elevated between 3 and 6 weeks and persisted up to 15 weeks. On the other hand a group of diabetic rats were normotensive with urinary protein excretion similar to controls and urinary kallikrein lower compared to control but significantly higher compared diabetic hypertensive rats. These data suggest that the association of progressive diabetic nephropathy with abnormal endothelium-dependent vasodilation may produce a high prevalence of hypertensive diabetes.


*Int. Jnl. Experimental Diab. Res.*, Vol. 2, pp. 195–199
Reprints available directly from the publisher
Photocopying permitted by license only

© 2001 OPA (Overseas Publishers Association) N.V.
Published by license under
the Harwood Academic Publishers imprint,
part of Gordon and Breach Publishing,
a member of the Taylor & Francis Group.
Printed in the U.S.A.

### The Diabetic Nephropathy and the Development of Hypertension in Rats

ADRIANA ZUCCOLLO[b], MONICA NAVARRO[b] and ORLANDO CATANZARO[a,*]

[a]*PROSIVAD-CONICET, Catedra de Animales de Laboratorio, Facultad de Farmacia y Bioquimica-UBA;*
[b]*PROSIVAD-CONICET, Instituto Universitario de Ciencias de la Salud. Facultad de Medicina-Fundacion Barcelo*

*(Received 1 May 2001; In final form 25 June 2001)*

**The present study was designed to examine the development of hypertension in diabetic rats treated with streptozotocin (STZ, 1mg/g bw). The rats were studied at 3, 6, 9, 12 and 15 weeks. From the third week the rats were divided in diabetic rats according their glycemias and controls, along 15 weeks. After the third week a group of rats showed increased urinary protein excretion (93, 134, 155 and 191%) compared to controls. In this group of rats the urinary kallikrein excretion was lower than control and the systolic blood pressure became significantly elevated between 3 and 6 weeks and persisted up to 15 weeks. On the other hand a group of diabetic rats were normotensive with urinary protein excretion similar to controls and urinary kallikrein lower compared to control but significantly higher compared diabetic hypertensive rats. These data suggest that the association of progressive diabetic nephropathy with abnormal endothelium-dependent vasodilation may produce a high prevalence of hypertensive diabetes.**

*Keywords*: Diabetes; Hypertension; Proteinuria; Kallikrein

### INTRODUCTION

Renal kallikrein is a senine protease located in the distal cortical nephron.[1] By its action on kininogen substrate it generates potent vasoactive kinins. Several observations link this enzyme and its product to the regulation of hemodynamic and ion transporting processes. Diabetes mellitus and hypertension are common chronic conditions which frequently coexist. According to Viberti *et al.*[2] the progression of diabetic nephropathy appears to be closely related to blood pressure elevation. Several studies show that the measure of urinary protein loss correlates with the rate of renal function deterioration. Early morphologic lesions of diabetic nephropathy develop in practically all IDDM within a few years after the onset of their metabolic abnormality.[3] On the other hand essential hypertension accounts for the majority of cases of hypertension in the diabetic population. Extensive evidence indicates an association of kallikrein-kinin system with blood pressure regulation. In the present study, we investigated whether the progressive increase of proteinuria and the decreased urinary kallikrein excretion may contribute to the hypertension in the diabetic state.

### MATERIALS AND METHODS

Neonatal male rats (2 days old) were used in his study. They were kept in a temperature controlled environment with standard laboratory food and water freely available. Diabetes was induced by a single subcutaneous injection of

*Address for correspondence: Universidad Del Salvador. Fac. de Medicina. Cat. de Fisiologia. Tucuman 1845 CP 1050 Buenos Aires, Argentina.

streptozotocin (STZ), 1mg/Kg bw dissolved in citrate buffer (pH 4.5). Control rats of the same age were injected with buffer alone. Blood samples were collected from the tail vein in subsequent periodic collections. Rats were housed two to three per cage, and had access to water and food ad libitum. Blood was collected from the tail vein and urinary samples were collected housing the animals in metabolic cages during 24h. Systolic blood pressure (SBP) was measured at different times with a pneumatic pulse transducer and occluding tail cuff (Grass Mod. 79 Poligraph, Grass Inst. Mass, USA). Rats were placed in restrainers at 28–30°C and after 1h, 4–6 determinations of SBP were made and average.

### Analytical Methods

Diabetes was confirmed by plasma glucose levels measured after STZ injection and at 21, 42, 63, 85, 105 days. Urinary protein was measured according to the procedure of Lowry *et al.*[4] and kallikrein was assayed as previously described.[5] These parameters were measured over a period of 15 weeks. Glomerular Filtration rate (GFR) was measured according to Henry *et al.*[6] Creatinine was measured with a Wiener kit reagent (Wiener Arg. SA).

### Chemicals

STZ was purchased from Sigma Chemical Co., St. Louis, Mo. It was dissolved in 0.02M citrate buffer, pH 4.5 and immediately injected intraperitoneal. The kallikrein assay was performed using the synthetic chromogenic substrate H-D-Val-Leu-Arg-pNA (S2266) from Kabi, Stockholm, Sweden. The para-nitroanilide (pNA) released by enzymatic reaction on the substrate was measured calorimetrically to determine kallikrein activity.[7]

### *Statistical Analysis*

Data are expressed as mean +/− SEM. Differences were assessed by ANOVA. Intergroup differences were analyzed by Student's test for unpaired data. Differences were considered significant at a level of $P < 0.05$.

### RESULTS

Three weeks after the initial STZ injection diabetic hypertensive rats (DH) showed significantly lower body weight compared to control rats. The average weight gain in DH rats was 2.33+/−0.27 *vs.* 3.14+/−0.31g/day. No differences were observed in diabetic normotensive (DN) compared to controls. After the 15th weeks these differences persisted and the average was 2.48+/−0.3 *vs.* 3.21+/−0.14g/day. The kidney/body weight ratio also, was significantly different at the 3rd and 15th weeks in DH rats. GFR was observed significantly reduced in DH rats compared to controls (Tab. I).

When the diabetic and control rats were studied at different periods of time the following results were obtained:

*Blood glucose levels*: Glucose steadily increased in one group of diabetic animals over time from

TABLE I Different parameters of the control and diabetic rats

| | 3rd week | | | 15 weeks | | |
|---|---|---|---|---|---|---|
| | Control | DN | DH | Control | DN | DH |
| | 10 | 12 | 12 | 9 | 10 | 11 |
| *Body weight (g)* | 330+/−16 | 302+/− 10 | 245+/−17* | 341+/−14 | 312+/−11 | 261+/−15* |
| *Kidney weight (g)* | 3.2+/−0.02 | 2.8+/− 0.5 | 2.9+/−0.4 | 3.1+/−0.4 | 3.8+/−0.6 | 4.7+/−0.2* |
| *Kidney weight* | | | | | | |
| *Body weight ratio %* | 0.97+/−0.04 | 0.93+/−0.03 | 1.18+/−0.08* | 0.90+/−0.02 | 1.19+/−0.04 | 1.88+/−0.07* |
| *GF R ul/min* | 0.37+/−0.05 | 0.50+/−0.04* | 0.38+/−0.06 | 0.39+/−0.05 | 0.45+/−0.02 | 0.28+/−0.07* |

DN: Diabetic Normotensive; DH: Diabetic Hypertensive.
Data are +/− DE *P<0.01 Diabetes *vs.* Control.

the 3rd week to 15th weeks(30, 48, 47, 46 and 49% respectively). On the other hand other group of diabetic rats showed 8, 25, 30 and 30% increased blood glucose over time (Fig. 1).

*Urinary protein excretion*: Urinary protein excretion was observed progressively increased over time in one group of diabetic rats (93, 134, 155, 199% DH) started from the 3rd week. In the DN rats the increase of urinary protein secretion was 25, 45, 50 and 57 from the 6th week (Fig. 2).

*Total Kallikrein excretion*: Kallikrein excretion was observed progressively decreased in DH rats from the 3rd to 15th weeks compared to controls. However in DN rats the urinary kallikrein excretion was significantly different from the 6th week compared to control (Fig. 3).

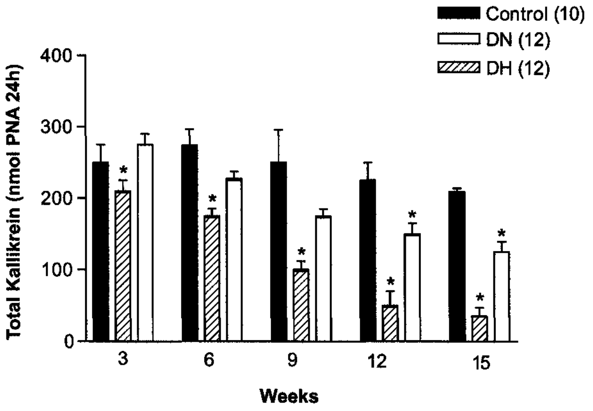

FIGURE 3 Urinary kallikrein excretion () Nr. of rats. Results are expressed as mean+/−SD. ANOVA was performed to evaluate differences between groups. *P>0.05 Control *vs.* DH and DN.

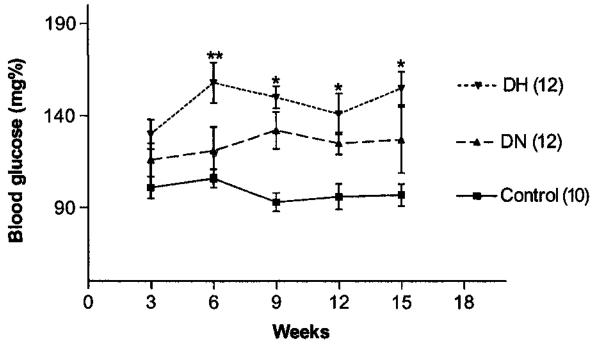

FIGURE 1 Blood glucose levels (mg%) in control, Diabetic Normotensive (DN) and Diabetic Hypertensive (DH). () number of rats per groups. Mean +/− SEM, *P<0.05; **P < 0.01.

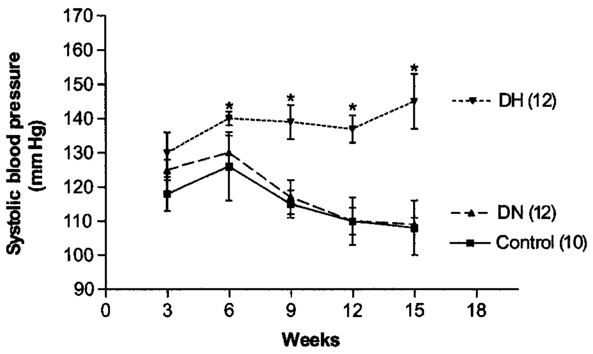

FIGURE 4 Systolic blood pressure in Control, Diabetic Normotensive (DN) and Diabetic Hypertensive (DH). () number of rats. Mean +/− SEM *P<0.01.

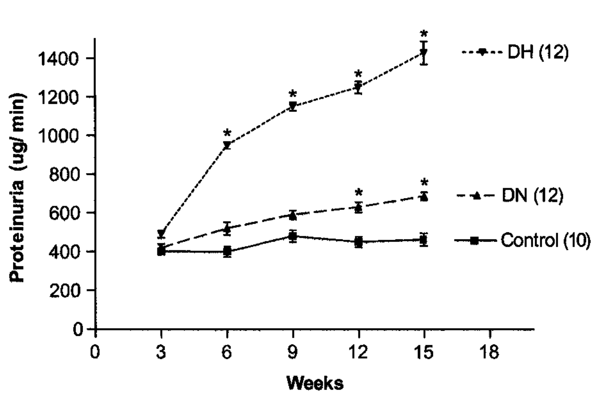

FIGURE 2 Urinary protein excretion in Control, Diabetic Normotensives (DN) and Diabetic Hypertensives (DH) rats. Mean +/− SEM *P<0.01.

*Systolic blood pressure*: SBP was studied in controls, DH and DN. In the DH rats the SBP was significantly increased along the experiment compared to controls rats (13, 18, 21, 27 and 32% respectively). On the other hand diabetic rats with moderated proteinuria and low kallikrein after the 6th week showed no significant differences of SBP *vs.* control rats (Fig. 4).

### DISCUSSION

The present study has clearly shown that increased urinary protein excretion and lower kallikrein secretion is, probably one of the possible mechanisms to produce diabetic hypertension. Marre *et al.*[8] suggested that microalbuminuria in some hypertensive NIDDM patients can be explained by the association between essential hypertension and NIDDM. Diagnosis of clinical nephropathy, however, is made by the appearance of persistent proteinuria which inaugurates a phase leading to an end-stage renal disease (ESDR). Thus hypertension is often associated with diabetic nephropathy.[9] Ours study examine the mechanisms responsible for the abnormalities in the renal function associated with the diabetic hypertension. We found that diabetes can produce some changes in the progressive increase of urinary protein excretion and reduced kallikrein secretion when blood glucose is persistently higher over time. Even though other mechanisms may contribute to reduce tubuloglomerular feedback and hyperfiltration in diabetes mellitus, stimulation of cotransporter by hSGK is likely to participate in the generation of diabetic hyperfiltration. Beyond that, stimulation of epithelial $Na^+$ channel and cotransporter is expected to induce $Na^+$ retention and thus favour the development of diabetic hypertensive disease.[10] In Fact, the prevalence of hypertension in this case correlates with the renal functional abnormalities and the duration of diabetes.[11] According to Christlieb [12] essential hypertension accounts for the majority of cases of hypertension in the diabetic population, and the pathogenesis of essential hypertension in diabetic patients is considered to be similar to that in non diabetic patients.[13] Furthermore an study by Knowler *et al.*[14] on Pima Indians showed that prediabetic elevated blood pressure occurred at higher rate (36%) in those patients with increased proteinuria and nephropathy than those with low or without proteinuria and nephropathy (12%) Interestingly diabetic rats with moderate hyperglycemia, low proteinuria and low kallikrein from the 9th week did not develop hypertension. The mechanisms trough which urinary protein excretion did not rise and kallikrein excretion was low in this group of DN rats is not completely clear. Previous studies have indicate that diabetic patients in poor control of their prior glycemias (HbA 1c 11%) had 177% of urinary protein excretion compared to controls, whereas diabetic patients with HbA 1c 11% showed less of 100% of urinary protein excretion. These results showed also that kallikrein secretion rate was correlated with the HbA 1c level.[15] The magnitude of the diabetes state produces an impairment of rat renal kallikrein synthesis with a decreased renal tissue and urinary kallikrein secretion as it was demonstrated by Jaffa *et al.*[16] in diabetic rats. On the other hand insulin treatment increased kallikrein mRNA levels, suggesting that diabetes suppresses kallikrein gene expression. Severely hyperglycemic diabetic rats with normal GFR had lower renal excretion of active kallikrein than control animals, whereas moderately hyperglycemia diabetic rats exhibited higher urinary levels of the enzyme.[15] According with our results in the group of rats with diabetic nephropathy the proteinuria was significant increased over the time with a correlation with decreased kallikrein. Taken together the data suggest that there is a defect in the expression and transcription of kallikrein in the diabetic kidney as it was postulated in diabetic rats by Jaffa *et al.*[16] Diabetic nephropathy produces structural abnormalities as hypertrophy of kidney, increase in the thickness of glomerular basement membranes, accumulation of extracellular matriz in the glomerulus, tubular atrophy and interticial fibrosis.[17] It is relevant to point out that functional alterations of diabetic nephropathy produces an early proteinuria and systemic hypertension.[18] In this regard, in human and animal diabetic models TGF-beta mRNA and protein levels are significantly increased in the glomeruli and tubulointerstitium.[17] On the other hand the deleterious effect of proteinuria on the progression of renal disease may be mediated *via* TGF-beta.[19] The suggestion that severely hyperglycemic diabetic rats with increased proteinuria and low kallikrein secretion shows hypertension is further supported by studies that shows that endothelium function is abnormal in diabetic rats.[20] In conclusion, the present study has found that in long-term experimental diabetes the progressive increase of urinary protein secretion in the early state of diabetes and low kallikrein excretion is one of the major pathways responsible of diabetic hypertension.

### *Acknowledgement*

This research was supported by a grant Nr. 4582 from the CONICET.

### *References*

[1] Vio, C. P. and Figueroa, C. D. (1983). Subcellular localization of renal kallikrein by ultrastructural immunocytochemistry. *Kidney Int.*, **28**, 36–42.
[2] Viberti, G. C. (1994). Prognostic significance of microalbuminuria. *Am. J. Hypertension*, **7**, 69s–72s.
[3] Osterby, R. (1983). Basement membrane morphology in diabetes mellitus. In: *Diabetes Mellitus: Theory and Practice* (3rd Edn.) edited by Ellemberg, M. and Rifkin, H., NY Medical Examination Publishing, pp. 323–341.
[4] Lowry, O. H., Rosebrough, N. J., Farr, A. L. and Randall, R. J. (1951). Protein measurement with the Folin reagent. *J. Biol. Chem.*, **193**, 265–275.
[5] Vila, S. B., Peluffo, V., Alvarado, C., Cresto, J. C., Zuccollo, A. and Catanzaro, O. L., *Agents and Actions*, **38**, 204–210.
[6] Henry, R. J., Cannon, D. C. and Winkilman, J. K. (1974). *Clinical chemistry principles and techniques*. Eds. Harper and Row, NY, pp. 541.
[7] Frieberger, P., Aurell, L. and Claeson, G. (1982). Chromogenic substrates for kallikrein and related enzymes. *Agents and Actions*, **9**, 83–90.
[8] Marre, M. and Fressinaud, P. (1990). Clinical effects of calcium antagonists in hypertensive diabetes. *J. of Card. Pharmacol.*, **16**(suppl. 2), 513–515.
[9] Reddi, A. S. and Camerini-Davalos, R. A. (1990). Diabetic nephropathy: An update. *Arch. Intern. Med.*, **150**, 31–42.
[10] Lang, F., Kingler, K. A., Wagner, C. A., Stegen, C., Warntges, S., Friedrich, B., Lanzerdorfer, M., Melzig, J., Moschem, I., Steuer, S., Waldegger, S., Sauter, M., Paulmichl, M., Gerke, V., Risler, T., Gamba, G., Capaso, G., Kandolf, R., Hebert, S. C., Massry, S. G. and Broer, S. (2000). Derange transcriptional regulation of cell-volume sensitive kinase h SGH in diabetic nephropathy. *Proc. Natl. Acad. Sci.*, USA, **97**, 8157–8162.
[11] Houston, M. C. (1989). Treatment of hypertension in diabetes mellitus. *Am. Heart Ass.*, **118**, 819–829.
[12] Christlieb, A. R. (1990). Treatment selection considerations for the hypertensive diabetic patients. *Arch. of Int. Med.*, **150**, 1167–1174.
[13] Mogensen, C. E. (1990). Managements of the diabetic patients with hypertension: Early screening and management, pp. 1717–1739, Raven Press Ltd. NY.
[14] Knowler, W. V., Bennet, P. H., Nelson, R. G., Saad, M. F. and Petit, D. J. (1988). Blood pressure before the onset of diabetes predicts albuminuria in Type 2 (non-insulin-dependent) diabetes. *Abs. Diabetology*, **31**, 509 a.
[15] Mayfield, R. K., Margolius, H. S., Bailey, G. S., Miller, D. H., Sens, D. A., Squires, J. and Namm, D. H. (1985). Urinary and renal tissue kallikrein in the STZ diabetic rats. *Diabetes*, **34**, 22–28.
[16] Jaffa, A. A., Velarde, V., Le Roith, D. and Mayfield, R. K. (1997). Induction of renal kallikrein and renin gene expression by insulin and IGF I in the diabetic rat. *Diabetes*, **46**, 2049–2056.
[17] Reeves, W. and Andreoli, T. E. (2000). Transforming growth factor B contributes to progressive diabetic nephropathy. *Proc. Natl. Acad. Sci.*, **97**, 7667–7669.
[18] Hostetter, T. H., Renke, H. G. and Brenner, B. M. (1982). The case for intrarenal hypertension in initiation and progression of diabetic and other glomerulopathies. *Am. J. Med.*, **72**, 375–380.
[19] Yamamoto, T., Takamura, T., Noble, N. A., Rouslahti, E. and Border, W. A. (1993). Expression of transforming growth factor beta is elevated in human and experimental diabetic nephropathy. *Proc. Natl. Acad. Sci.*, **90**, 1814–1818.
[20] Costa, M. A., Balaszczuk, A. M., Domingues, A., Catanzaro, O. L. and Arranz, C. (1998). Effects of L_NAME and L-Arg on arterial blood pressure in normotensive and hypertensive streptozotocin diabetic rats. *Acta Physiol. Pharmacol. Latinoam.*, **48**, 59–63.

